# Digital orthodontic setup and clear aligners system for treating adult patients with periodontitis: a descriptive case report

**DOI:** 10.3389/fdmed.2024.1353114

**Published:** 2024-02-05

**Authors:** Vincenzo Ronsivalle, Claudia Malara, Marco Cicciù, Piero Venezia, Antonino Lo Giudice

**Affiliations:** ^1^ Department of General Surgery and Medical-Surgical Specialties, Dental Clinic - Unit of Oral Surgery, University of Catania, Catania, Italy; ^2^ Department of General Surgery and Medical-Surgical Specialties, Dental Clinic - Unit of Orthodontics, University of Catania, Catania, Italy

**Keywords:** digital setup, clear aligners, CAT, digital orthodontic, periodontitis, orthodontic treatment

## Abstract

This case report describes the treatment of an adult female patient with a history of periodontitis (Stage 3 -Grade B) and presenting significant crowding in both maxillary and mandibular arches. After periodontal stabilization, the patient underwent orthodontic treatment with clear aligners (CAT) for less than one year. CAT provided an effective quantitative and qualitative control of the forces applied to resolve the malocclusion. This case report provides a detailed description of the clinical strategy and features associated with the clear aligners system which were considered ideal tools for treating periodontal patients during the decisional workflow. The orthodontic treatment goals have been successfully achieved considering functional, periodontal, and aesthetic expectations. Several characteristics of clear aligners could support the recurrence of this orthodontic system for treating patients with a history of periodontitis or at risk of periodontal damage.

## 1 Introduction

Malocclusions in adults are often correlated with the clinical condition of periodontitis; this is due both to the incidence of periodontal disease which progressively increases with age (high as 76%–92%) (1), and to direct effects on occlusion that occur following the loss of periodontal support (2). Malocclusion can also contribute to the progression of periodontal disease in adult subjects over time; indeed, crowding is associated with an increase in plaque index and occlusal trauma with negative effects on the alveolar bone levels (3). For this reason, orthodontic treatment has been claimed as a treatment option to improve oral hygiene and remove those factors causing occlusal trauma (4).

Although periodontitis is not considered an absolute contraindication to orthodontic treatment in adult patients (5).

Caution has been advocated in treating patients with periodontal disease since the reduction of alveolar bone level causes the center of rotation to be located more apically, increasing the moment of forces expressed and torque (6); also, the rate of alveolar bone resorption is greater than that occurring under orthodontic forces (2, 7, 8). For these reasons, adult patients with periodontitis should be treated with the appropriate mechanics and with light force to maintain the health and stability of the periodontal environment (5). Clear aligner therapy is often the first choice for orthodontic treatment in adults due to the high patients’ aesthetic demands, comfort, and better control of oral hygiene (9). The possibility to define the biomechanics “*a priori*” and to stage the planned orthodontic movements would represent another advantage of clear aligners, especially in the management of patients affected by periodontal disease. In this regard, the present manuscript aims to describe the clinical strategy that led to a combined ortho-perio treatment performed with clear aligners, from the diagnosis and treatment plan to the clinical execution. In particular, the present case report describes the treatment of an adult female affected by periodontitis and presenting dental crowding in both maxillary and mandibular arches.

## 2 Case report

The 50-year-old patient with a diagnosis of stage 3 grade B periodontitis was referred to an Orthodontic Private Practice in Catania for establishing a multidisciplinary treatment approach. The chief complaint was to correct crowding in the anterior mandibular region to improve smile aesthetics and for better maintenance of oral hygiene.

**Figure 1:**
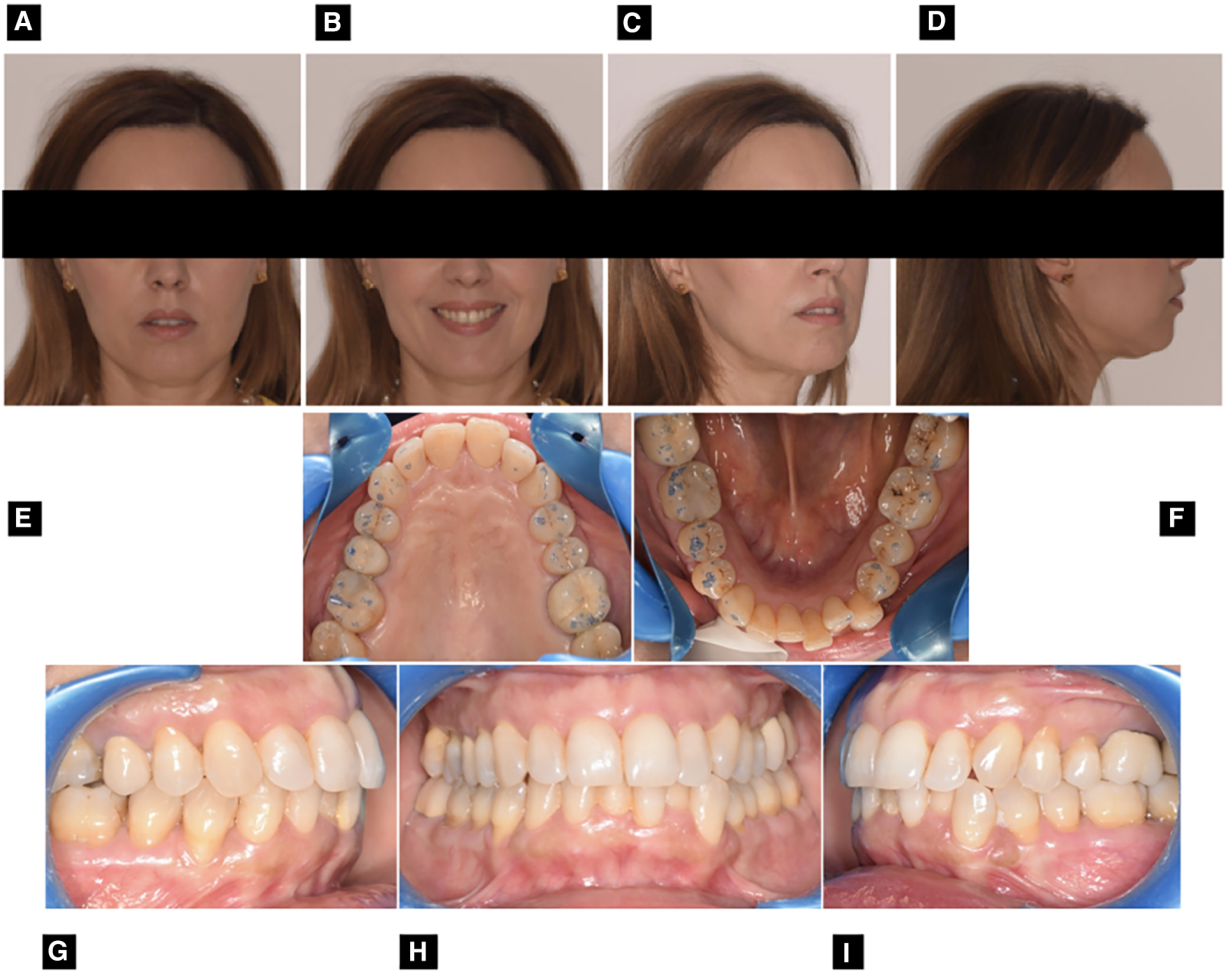
Pre-treatment extraoral and intraoral photographs: frontal view with lips at rest (**A**) frontal view with smile (**B**) ¾ view (**C**) right profile view (**D**) upper occlusal view (**E**) lower occlusal view (**F**) right Side view (**G**) frontal view (**H**) left side view (**I**).

**Figure 2:**
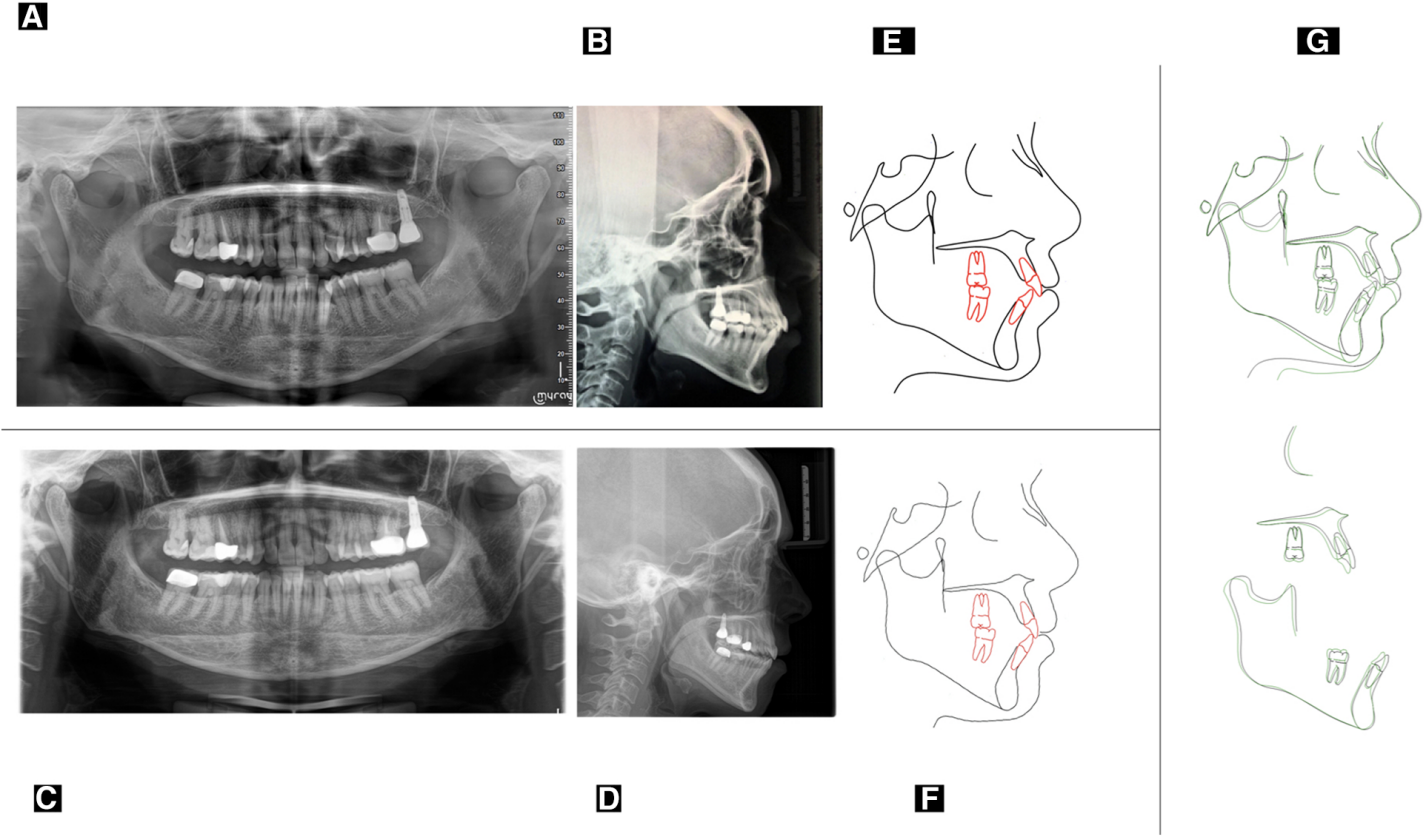
Initial panoramic radiographs (**A**) initial latero-lateral teleradiography (**B**) final panoramic radiographs (**C**) final latero-lateral teleradiography (**D**) pre-treatment cephalometric tracing (**E**) post-treatment cephalometric tracing (**F**) cephalometric superimposition between pre-treatment (black) and post-treatment (green) cephalograms (**G**).

The extra-oral examination, performed via clinical analysis (static and dynamic) and photographic evaluation, revealed a eugnathic profile associated with a slight bi-maxillary protrusion, resulting in lip incompetence at rest (Figures 1C,D) From the frontal view, it was possible to note the gingival exposure during smiling and the presence of narrowed buccal corridors (Figures 1A,B). The intra-oral examination (Figures 1E–I), performed via clinical intra-oral inspection and analysis of digital models, revealed mild contraction of both dental arches, with severe crowding in the anterior mandibular region associated with ectopia of the tooth 3.3. (Figure 1I). Traumatic occlusal forces resulting from crowded anterior teeth and linguo-inclined lower posterior teeth were retrieved and detected using articular paper (Figure 1E,F). The patients presented bilateral molar and canine class I (Figure 1G,I) with overjet values of 4 mm and overbite of 3 mm. There is also a deviation of the lower midline to the right (Figure 1H). Cephalometric examination revealed a Class II skeletal sagittal pattern associated with a tendency to hyper-divergency of the mandibular plane, slight proclination of the upper incisor, increased proclination and compensation of the lower incisor (Figure 2B and Table 1). The panorex revealed a generalized pathological migration of the alveolar bone crest in both maxillary and mandibular arches while no signs of periapical inflammation were reported (Figure 2A).

**Table 1:** Cephalometric parameters recorded before and after treatment.

Sagittal skeletal relations			
Measurement	Norm	Pre treatment	Post treatment
Maxillary position S-N-A	82° ± 3,5°	78,9°	76,9°
Mandibular position S-N-Pg	80° ± 3,5°	74,8°	74,2°
Sagittal jaw relation A-N-Pg	2° ± 2,5°	3,9°	2,8°
Vertical skeletal relations			
Maxillary inclination S-N/ANS-PNS	8° ± 3,0°	10°	6,6°
Mandibular inclination S-N/Go-Gn	33° ± 2,5°	36,5°	39,4°
Vertical jaw relation ANS-PNS/Go-Gn	25° ± 6,0°	28,8°	36,9°
Dento-basal relations			
Maxillary incisor inclination 1-ANS-PNS	110° ± 6°	111,7°	102°
Mandibular incisor inclination 1-Go-Gn	94° ± 7°	102,9°	94,5°
Mandibular incisor compensation 1-A-Pg (mm)	2 ± 2 mm	5 mm	5 mm
Dental relations			
Overjet (mm)	3,5 ± 2,5 mm	4,2 mm	3 mm
Overbite (mm)	2,5 ± 2,5 mm	3,4 mm	1 mm
Interincisal angle 1/1	132° ± 6,0°	119,8°	130°

The periodontal analysis confirmed a chronic periodontal disease of Stage 3—Grade B. The patient presented generalized periodontal pockets mostly remarkable on the lower arch. The following periodontal indices were measured and reported in the periodontal chart: Periodontal Pocket Probing Depth: PPD > 4 mm = 20; Probing depth: mean PD = 4.5 mm; Gingival bleeding on probing: BOP = 25%; Plaque index (PI) = 40%. The most significant periodontal pockets were as follows: 3.6 DV = 10.0 mm, 3.4 MV = 7.00 mm, 4.6 DV = 8 mm, 3.1 DV = 5 mm, 4.1 MP = 5.5 mm, 4.2 DP = 5.5 mm, 2.1 DP = 5.0 mm, 1.1 MV = 6 mm, 1.2 DV = 5,0 mm, 1.6 MP = 7 mm. Microbiological examination revealed high concentrations of Porphyromonas gingivalis and Tannerella forsythia.

### 2.1 Multidisciplinary treatment plan

A multi-disciplinary treatment was planned and managed in two phases, involving periodontal therapy and stabilization (stage 1) and orthodontic treatment with aligners (stage 2).

#### 2.1.1 Periodontal treatment (stage 1)

In the first phase, the non-surgical causal treatment of the periodontal pockets was carried out. The goal was to start the orthodontic treatment once a value of BOP <10% and PI <20% was reached (5). The treatment process consisted of a full-mouth scaling and root planing (SRP) subdivided into two separate sessions on 2 consecutive days. Two right quadrants were instrumented during the morning session and the two left ones in the afternoon session. Treatments were performed under local anesthesia. SRP was performed using both hand and ultrasonic instrumentation by tips No. 5/6/7, which was used with constant water irrigation with a 20.000 Hz frequency. At the end of treatment, the patient was instructed and motivated to perform personal oral hygiene. Six months after completion of the periodontal treatment, a clinical examination was performed, and a new periodontal chart was generated. The values recorded were: BOP 9%, PI 17%, PPD >4 mm = 3. The most significant pre-treatment periodontal pockets, at the end of stage 1 were as follows 3.6 DV = 8.0 mm, 3.4 MV = 4 mm, 4.6 DV = 4,5 mm, 3.1 DV = 3 mm, 4.1 MP = 3,5 mm, 4.2 DP = 3,5 mm, 2.1 DP = 3,5 mm, 1.1 MV = 4 mm, 1.2 DV = 3,0 mm, 1.6 MP = 4 mm.

#### 2.1.2 Orthodontic treatment with clear aligners (stage 2)

**Figure 3:**
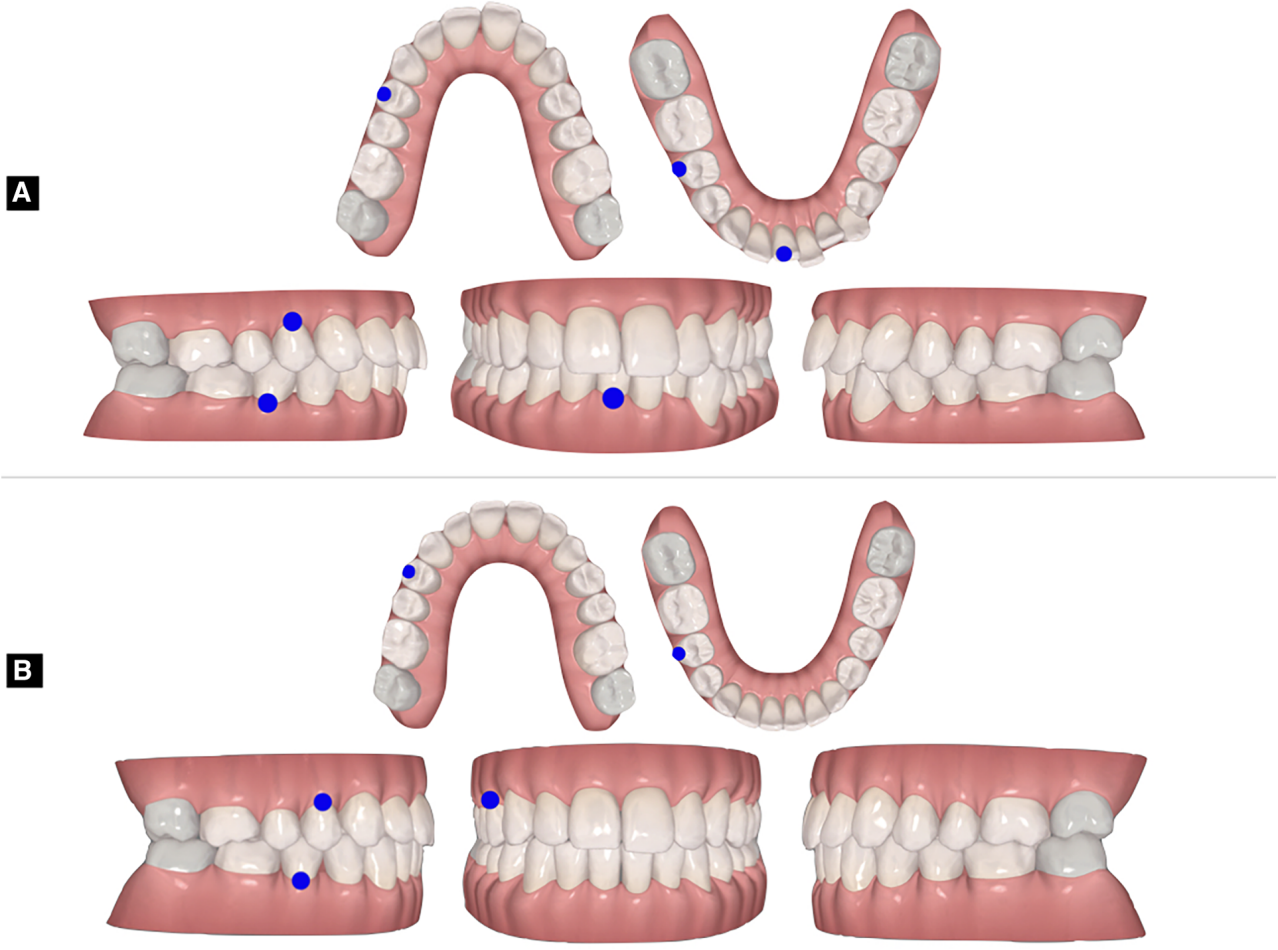
Orthodontic setup approved in the clinCheck software: baseline models (**A**) and final virtual occlusion (**B**).

Once the periodontal condition was improved, the patient was re-evaluated to define the objectives of the orthodontic treatment. The maxillary and mandibular arch impression and bite registration were recorded using the intraoral scanner. The impressions were sent to Invisalign® to plan the treatment with clear aligners using the digital platform ClinCheck® software (Figures 3A,B). The main goals identified were: to resolve crowding, obtain midline coincidence, maintain baseline molar Class, normalize overbite and overjet, eliminate strong occlusal contact, normalize the Bolton Ratio, enhance smile aesthetics and gingival exposure, avoid worsening of periodontal support.

### 2.2 Treatment options

An alternative treatment strategy for this patient was the extraction of teeth 14, 24, 34, and 44. This therapeutic option simplified the alignment biomechanics, as the first premolars were the teeth proximal to the crowding, considering the ectopic position of teeth 33 (vestibular displacement). Also, teeth 14, 24, 34, and 44 were mostly affected by gingival recessions. However, this approach would have entailed potential negative effects on lip support and soft tissue-related smile attractiveness. Also, this approach would have significantly extended the overall treatment time.

The second alternative treatment strategy was the extraction of the teeth 31. This therapeutic option simplified the alignment biomechanics providing immediate space in the crowded area, avoiding remarkable repercussions on lip support and soft tissue-related smile attractiveness compared to the previous treatment option. However, this approach would have determined an alteration of the Bolton Ratio with an increase of overjet and negative repercussions on periodontal tissues in terms of occlusal stability.

The third alternative treatment strategy was the application of interproximal reduction (IPR) associated with slight posterior arch expansion to gain space for arch alignment (10, 11). This treatment option would provide the maintenance of normal overjet and overbite, avoiding tooth/treatment extraction and reducing the overall treatment timing. However, it would require better control of the intensity of force application to avoid a significant overload over the bony periodontal support.

A diagnostic setup was performed for all three therapeutic options to help the clinician choose the most appropriate treatment. After being processed, all diagnostic set-ups were also proposed to the patient to help her better understand the treatment's goals and the different ways to achieve them.

The patient agreed to the third alternative treatment strategy.

### 2.3 Treatment progress

The biomechanics used were planned to resolve the malocclusion in relation to the periodontal tissues were defined in the clincheck® software (Figures 3A,B):

• Regularization of the shape of the upper and lower arches by slight transversal expansion
• IPR 22-23-24 (Programmed: 0.8 mm; Clinically performed: 60%)
• IPR 33-43: (Programmed: 1.4 mm; Clinically performed: 70%)
• No movement by the 7 s
• Intrusion of lower incisors
• Extrusion of upper incisors
• Programmed movement: 0.15 mm for the aligner, to avoid less stress on the dentition and the alveolar ridge crest.
• Attachments were posteriorly placed providing anchorage for vertical movements of the anterior teeth.

Optimized attachments have been chosen to favor the canine’s rotation.

The compliance requested was 22 h every day; the patient was instructed to move on to the next aligner after 14 days. The treatment plan included 18 maxillary aligners and 14 mandibular ones. At the end of the first phase, a refinement ClinCheck® was elaborated (5 additional aligners).

## 3 Results

**Figure 4:**
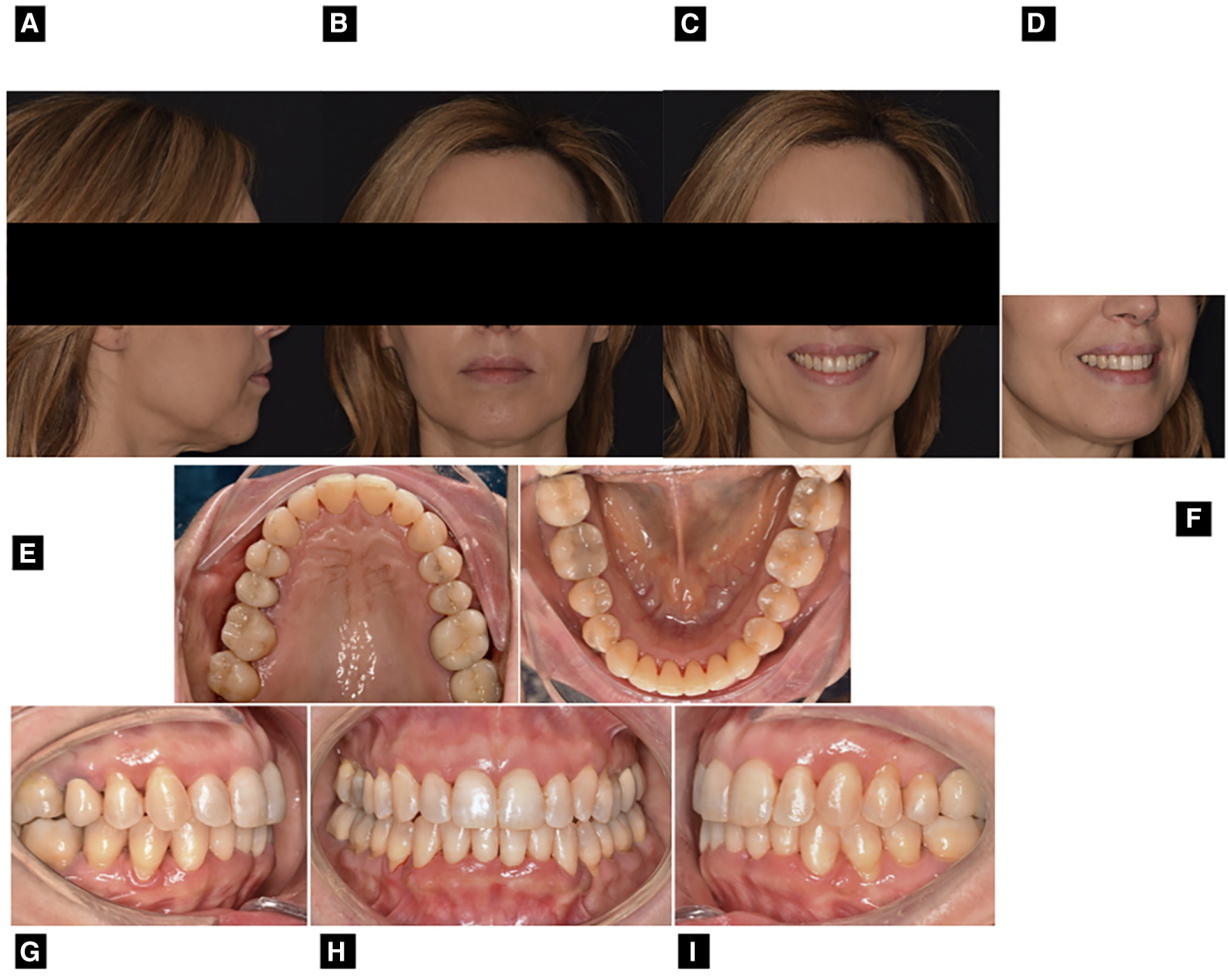
Post-treatment extraoral and intraoral photographs: right profile view (**A**) frontal view with lips at rest (**B**) frontal view with smile (**C**) ¾ view (**D**) upper occlusal view (**E**) lower occlusal view (**F**) right Side view (**G**) frontal view (**H**) left side view (**I**).

The orthodontic treatment goals have been successfully achieved, in almost one year of therapy (overall orthodontic treatment time) (Figure 4A–I). Strong occlusal contacts were eliminated, preventing traumatic occlusion and the risk of aggravating periodontal damage in the future (9). The inclination of the upper and lower incisors improved, while the anterior limit of the lower dentition maintained the same value as the baseline (Figure 2D). Also, there was an improvement in lip competence due to the enhanced position of the upper and lower incisors (12–14). A clear representation of post-treatment changes is shown by the superimposition between pre-treatment and post-treatment cephalograms (Figures 2E–G and Table 1) (15). Finally, there was a better smile consonance according to the smile arc and better gingival exposure (Figure 4D). The radicular parallelism was appropriate (Figure 2C). The periodontal examination revealed the absence of orthodontic mobility and the absence of post-treatment active periodontal pockets. The following periodontal parameters were recorded at the end of the orthodontic treatment:

BOP 9%, PI 16%, PPD >4 mm = 3. The most significant pre-treatment periodontal pockets, at the end of stage 1 were as follows 3.6 DV = 8.0 mm, 3.4 MV = 4 mm, 4.6 DV = 4,5 mm, 3.1 DV = 2,5 mm, 4.1 MP = 3,5 mm, 4.2 DP = 3,5 mm, 2.1 DP = 3,0 mm, 1.1 MV = 4,5 mm, 1.2 DV = 3,0 mm, 1.6 MP = 4 mm.

A removable vacuum-formed retainer was given to the patient to maintain the result achieved and the patient was asked to wear the appliance at night. Although fixed retainers represent a valid solution to maintain the position of mandibular incisors in the long term in subjects with a risk of recrudescence of periodontitis (16), they are shown to be associated with more plaque accumulation compared to removable retainers (17).

## 4 Discussion

The main issue was retrieving space for resolving the severe crowding in the anterior mandibular region and the ectopia of the tooth 3.3, without aggravating the periodontal support and the position of the lower incisors (18, 19). We opted for CAT since this system allows us to plan the necessary amount of IPR and monitor the inter-proximal space closure consistently with the digital treatment plan for tooth movement. Convincing evidence confirms that the IPR planned in the digital setup could overestimate the space necessary to resolve dental crowding (20, 21). In this case, the patient's malocclusion was treated by applying 60%–70% of the planned IPR and monitoring the treatment progress with a consistent appointment schedule. The authors are conscious that this concept is in contrast with recently advocated technology based on recurrent usage of telemedicine to reduce the number of visits at the chairs-side (22).

Another advantage of CAT is that there are minor differences between the positions of the teeth intraorally and the positions of the teeth planned in the aligner (this position will be reached using the pushing force applied by the aligners itself). Since the planned amount of stripping for each aligner is “included” in the free space between the tooth and the aligner (23, 24) the combination of pushing forces and planned stripping provides a better control of tooth movement, preserving the anterior limit of the dentition. Such intrinsic characteristics would represent an absolute indication for treating anterior crowding in periodontal patients (25).

Maintaining the anchorage is quite challenging in the presence of compromised periodontium, regardless of the type of appliance used (2). In this case, we enhanced posterior anchorage by incorporating three applications offered by clear aligners: (1) defining movement priority = we prevented multiple simultaneous movements by prioritizing posterior expansion before alignment; (2) overengineering = we asked for additional distal crown-tip in the molar and premolar region to offset the mesial inclination that may have occurred during the correction of incisor torque; (3) attachments = the anchorage effect was ensured through the application of efficient rectangular attachments, contributing to improved control over root movements.

With CAT is possible to customize the biomechanics by staging tooth movements which allows modulating the orthodontic forces from qualitative and quantitative perspectives. Furthermore, the rate of tooth movement may also be adjusted according to the individual's bone physiology and by altering the scheduled number of days for aligner changes, depending on the individual's response to tooth movement (23).

Establishing the appropriate staging protocol is recommended in subjects with an increased risk of periodontal damage to reduce the stress on the teeth and the strain at the top of the alveolar crest. This is extremely important with clear aligners since they exert instantaneous stress on the dentition that is 50 to 500 times that expressed by the continuous light force of NiTi archwire using fixed appliances (26).

While the standard displacement set for each clear aligner is generally set at 0.20–0.30 mm/2° per step (27, 28), a range between 0.10 mm and 0.18 mm and 1° was recommended in the presence of incisor's proclination or periodontal damage to avoid strain on the alveolar ridge crest. In particular, 0.15 mm seems to be the optimal displacement for patients with periodontitis in class III (29). In this regard, we opted for a slower movement stage (0,15 mm/1°) along with specific design features (see below) to obtain good periodontal results (26, 30).

Clear aligners are claimed as a better solution for maintaining periodontal health conditions compared to fixed appliances, due to better management of daily oral hygiene maneuvers (31). The patient found it beneficial to be able to remove the aligners and maintain her oral hygiene routine without the encumbrance of fixed components of the conventional orthodontic appliance. Also, clear aligners cover most of the crown, hindering the accumulation of dental plaque on the teeth and preventing the migration of the plaque from supragingival dental plaque to subgingival tissues, reducing the risk of potential damage to the teeth (32). On the other side, evidence would suggest that the extensive day-long coverage of tooth surfaces promotes the accumulation of soft matter, potentially giving rise to sub-chronic inflammation (33, 34), and that the permanent coverage of tooth surfaces may disrupt the natural cleansing effect of saliva on dental tissues (35, 36).

Therefore, the actual literature emphasizes the paramount importance of oral hygiene practices rather than the choice of the type of orthodontic appliance in maintaining a healthy periodontal status since fixed appliances and clear aligners are exposed to different types of risk for periodontal damage. From the clinical perspective, this means that patients’ attitudes to oral hygiene and lifestyle should also be considered for this choice (18, 37). With this notion in mind, clear aligners well integrate with the higher rate of cooperation reported in adults and could represent the first-line option in periodontal patients, compared to fixed appliances.

Finally, another benefit of clear aligners system is the possibility of establishing and visualizing treatment outcomes using a digital setup (38). In truth, with clear aligners, clinicians are “obligated” to study and plan “*a priori*” the treatment strategy and the appropriate bio-mechanics in the digital platform, generating a more disciplined “proactive” approach compared to the decision-making process used with fixed appliance systems (39, 40). At the beginning, clinicians can request a diagnostic set-up including different treatment options that are evaluated in terms of length of time in treatment, degree of difficulty of tooth movements, optimization of tooth size/arch length discrepancies, occlusal result, and aesthetic outcomes (41, 42). After diagnostic evaluation, careful planning of the orthodontic set-up allows for the optimization of the bio-mechanics strategy and the appropriate virtual treatment plan design, to increase the system's predictability in periodontal patients (43). The orthodontic set-up represents also an excellent clinical tool to improve communication with the patient since it allows one to visualize the aesthetic expectations and, at the same time, to improve the perceptions of the treatment process and outcomes. Being more conscious and confident with the treatment experience, satisfaction of treatment outcomes increases (44–46). In this regard, the patient was satisfied with the results achieved that she perceived as consistent with the virtual diagnostic setup illustrated before treatment.

Finally, concerning treatment experience, the patient showed excellent compliance with the aligners; no pain, mucosal irritations, aesthetic, or speech problems were reported during the orthodontic treatment. The quality of life during treatment was judged as high since the treatment was comfortable and compatible with her active social life (47).

## 5 Conclusions

The present case report describes how a well-codified clinical strategy and combined multidisciplinary approach can lead to successful treatment outcomes in an adult periodontal patient. Several characteristics of clear aligners could support the recurrence of this type of appliance for managing orthodontic treatment in patients with a history of periodontitis or at risk of periodontal damage.

## Data Availability

The original contributions presented in the study are included in the article/Supplementary Material, further inquiries can be directed to the corresponding author.
